# Non‐enzymatic cross‐linking of collagen type II fibrils is tuned via osmolality switch

**DOI:** 10.1002/jor.23857

**Published:** 2018-02-13

**Authors:** Behdad Pouran, Parisa R. Moshtagh, Vahid Arbabi, Jessica Snabel, Reinout Stoop, Jeffrey Ruberti, Jos Malda, Amir A. Zadpoor, Harrie Weinans

**Affiliations:** ^1^ Department of Orthopedics UMC Utrecht Heidelberglaan100, 3584CX Utrecht The Netherlands; ^2^ Faculty of Mechanical, Maritime, and Materials Engineering, Department of Biomechanical Engineering Delft University of Technology (TU Delft) Mekelweg 2, 2628CD Delft The Netherlands; ^3^ Faculty of Engineering, Department of Mechanical Engineering University of Birjand 615/97175 Birjand Iran; ^4^ Department of Metabolic Health Research TNO P.O. Box 2215 2301 CE Leiden The Netherlands; ^5^ Department of Bioengineering Northeastern, University 360 Huntington Avenue Boston Massachusetts 02115; ^6^ Faculty of Veterinary Sciences, Department of Equine Sciences Utrecht University Yalelaan 112 3584 CM Utrecht The Netherlands; ^7^ Department of Rheumatology UMC Utrecht Heidelberglaan100 3584CX Utrecht The Netherlands

**Keywords:** cartilage ageing, collagen fibrils, micro‐indentation, glycation, micro‐CT, pentosidine level

## Abstract

An important aspect in cartilage ageing is accumulation of advanced glycation end products (AGEs) after exposure to sugars. Advanced glycation results in cross‐links formation between the collagen fibrils in articular cartilage, hampering their flexibility and making cartilage more brittle. In the current study, we investigate whether collagen cross‐linking after exposure to sugars depends on the stretching condition of the collagen fibrils. Healthy equine cartilage specimens were exposed to l‐threose sugar and placed in hypo‐, iso‐, or hyper‐osmolal conditions that expanded or shrank the tissue and changed the 3D conformation of collagen fibrils. We applied micro‐indentation tests, contrast enhanced micro‐computed tomography, biochemical measurement of pentosidine cross‐links, and cartilage surface color analysis to assess the effects of advanced glycation cross‐linking under these different conditions. Swelling of extracellular matrix due to hypo‐osmolality made cartilage less susceptible to advanced glycation, namely, the increase in effective Young's modulus was approximately 80% lower in hypo‐osmolality compared to hyper‐osmolality and pentosidine content per collagen was 47% lower. These results indicate that healthy levels of glycosaminoglycans not only keep cartilage stiffness at appropriate levels by swelling and pre‐stressed collagen fibrils, but also protect collagen fibrils from adverse effects of advanced glycation. These findings highlight the fact that collagen fibrils and therefore cartilage can be protected from further advanced glycation (“ageing”) by maintaining the joint environment at sufficiently low osmolality. Understanding of mechanochemistry of collagen fibrils provided here might evoke potential ageing prohibiting strategies against cartilage deterioration. © 2018 The Authors. *Journal of Orthopaedic Research* Published by Wiley Periodicals, Inc. on behalf of Orthopaedic Research Society. J Orthop Res 36:1929–1936, 2018.

In normal articular cartilage, the extracellular matrix (ECM) includes structured collagen type II fibrils, depth‐wise distributed glycosaminoglycan macromolecules (GAGs), and interstitial water.^1^ In osteoarthritis (OA), proteolytic activity initiates marked changes in the ECM of cartilage.^2^ The depletion of GAGs is often considered as early signs of OA, whereas irreversible degradation of collagen fibrils can be observed in the developing stages of OA followed by macroscopic degeneration of ECM in the late stage OA.^3^, ^4^ Contrary to OA, normal ageing does often not affect the major constituents, nor the organization of ECM, but rather contributes to formation and accumulation of so‐called advanced glycation end‐products (AGEs) including pentosidine cross‐links, which chemically affect the collagen molecules.^5^ The AGEs are formed by a chain of irreversible reactions between two adjacent amino acids of Arginin and Lysin found as the repeating units in the triple‐helix tropocollagen molecules.^6^ Not only is their production augmented due to increased systemic sugar levels, for example, in diabetes, but is also observed in the turnover processes of the ECM.^5^ Apart from obvious biomechanical effects of AGEs due to the impaired function of the collagen fibrils and increased brittleness,^5^ at the cellular level they interact with the surface receptors of chondrocytes.^5^ In short, AGEs stimulate catabolic pathways leading to upregulation of matrix metalloproteinases (MMPs) that are responsible for degrading the ECM components.

The three‐dimensional (3D) spatial orientation of collagen either in single molecular state or fibrillar state has been shown to affect enzymatic degradation of collagen.^7^, ^8^, ^9^ There is some evidence that stretching of collagen fibrils decelerates MMPs enzymatic activity.^7^, ^9^, ^10^, ^11^ This could be due to the 3D orientation of the amino acids that form the cleavage sites in the backbone of collagen fibrils, which might only interact with enzymes in a specific (low stretched) conformation. Similarly, the essential amino acids involved in advanced glycation, namely arginine and lysin,^12^ may need a specific 3D conformation to interact with sugars and create cross‐links.

External osmotic pressure of the solution to which cartilage is exposed controls its swelling state due to the in‐/out‐flux of water.^13^, ^14^, ^15^ Articular cartilage loses water when exposed to hyper‐osmolar solution, gains water when exposed to hypo‐osmolar solution, while exposure to iso‐osmolar solution is not expected to alter its shape. Here, we investigate the effects of osmotically driven mechanical conditioning of collagen fibrils within equine articular cartilage samples on their chemical response to the non‐enzymatic cross‐linking, in other words “artificial ageing.” This “artificial ageing” is induced by using l‐threose as the glycating agent under various osmotic conditions of hypo‐, normal, and hyper‐osmoality, thereby creating water outflow or inflow^14^ and consequent shrinkage or stretching of the collagen fibers. The micro‐scale effective elastic modulus, pentosidine level, surface color, and fixed charge density were then characterized to determine whether or not osmotic stretching of collagen fibrils protects them against non‐enzymatic glycation.

## MATERIALS AND METHODS

### Sample

**Figure 1:**
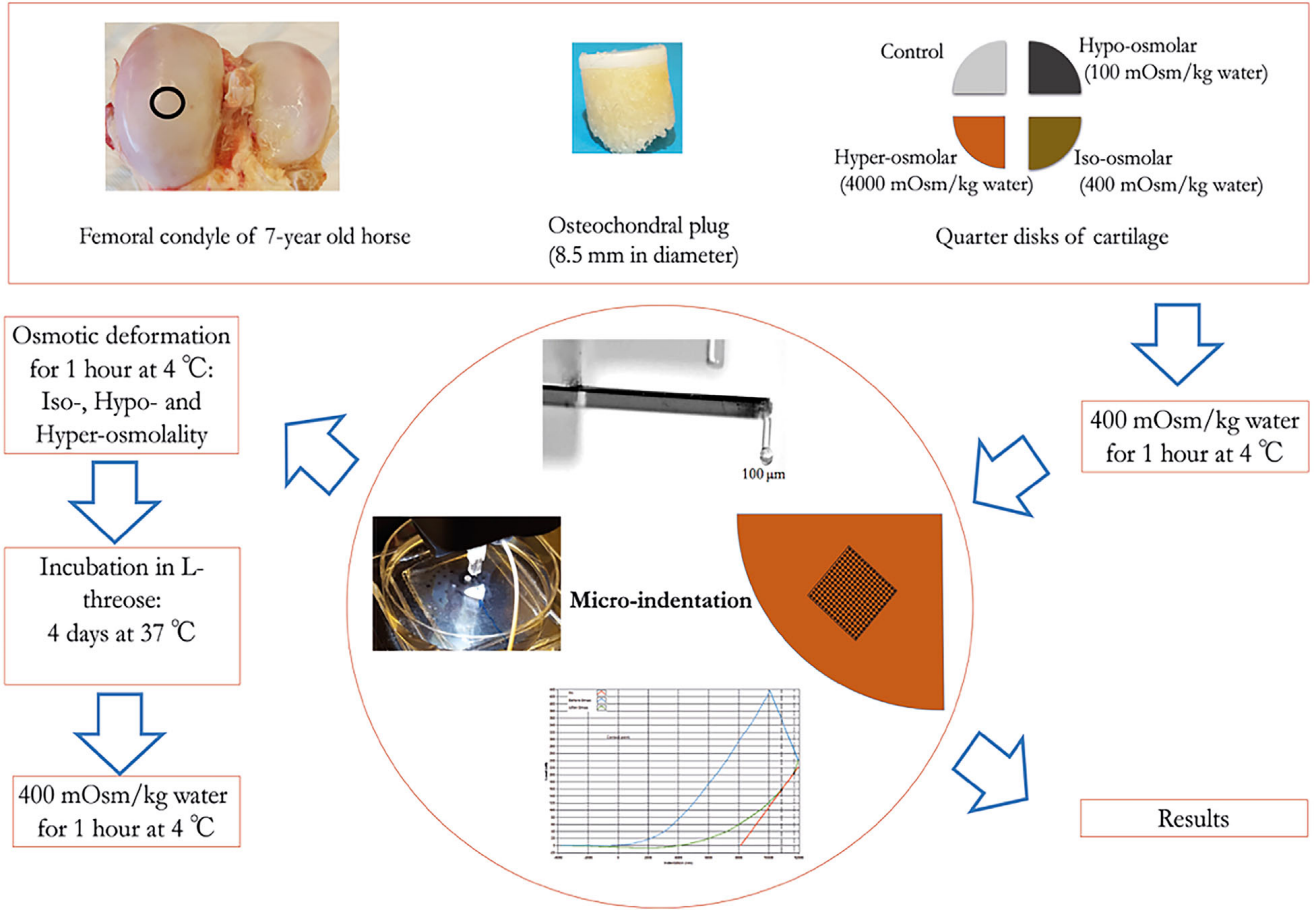
Schematic of the osmotic deformation, l‐threose incubation and micro‐indentation experiments. Osteochondral plugs are extracted from Equine femoral condyle of 7‐year‐old horse using custom‐made drill bits. Full thickness of cartilage is then removed from the subchondral bone using razor blade and split into four quarter disks. Three disks undergo their corresponding osmotic deformations, whereas one serves as control negative (no incubation with l‐threose). The cartilage specimens are equilibrated at 4°C in a 400 mOsm/kg water bath and therefore are prepared for the initial micro‐indentation tests. Matrix micro‐indentation is performed on each specimen in an area of 1.5 mm × 1.5 mm containing 81 equally spaced indentation points. Thereafter, samples are treated osmotically corresponding to our desired conditions and then l‐threose (50 mM) is added to each solution. Following the l‐threose incubation at 37°C, specimens are equilibrated in 400 mOsm/kg water for an hour before final matrix micro‐indentation. The change of the effective Young's modulus is reported as the result.

Equine osteochondral plugs (*n *= 5, *φ* = 8.5 mm) were extracted from visually‐intact femoral condyle (∼7 years old) using a custom‐made drill bit while care was taken to avoid overheating of the drilling site by constantly spraying phosphate buffer solution (PBS, Gibco, UK). Post‐extraction, the samples were stored at −20°C until further use. The full cartilage layer (∼2mm) was then carefully cut from the osteochondral plug followed by splitting it into four quarter disks. Each quarter disk was then equilibrated in a separate solution containing the required amount of NaCl and protease inhibitors (cOmplete, Roche, Mannheim, Germany) at an osmolality of 400 mOsm/kg water (0.2 M NaCl) at room temperature before performing micro‐indentation tests (Fig. 1).

### Mechanical Conditioning

Four‐quarter disks were obtained from each osteochondral plug, where each underwent different mechanical conditioning by adjusting the osmotic pressure of the external bath to hypo‐ (100 mOsm/kg water or 0.05 M), iso‐ (400 mOsm/kg water or 0.2 M) and hyper‐ (4,000 mOsm/kg water or 2 M) osmolalities using NaCl. Prior to incubation with l‐threose (50 mM, Sigma–Aldrich, Slovakia), each quarter disk was placed in its respective solution (2 ml), that is, hypo‐, iso‐, or hyper‐osmolal, for one hour (pre‐conditioning of collagen fibrils). Then, each quarter disk was transferred to 50 mM l‐threose solution having the same osmolality as in the pre‐conditioning step, except one quarter disk which functions as a non‐l‐threose control sample (400 mOsm/kg). Two quarter disks under hypo‐osmolality and two quarter disks under hyper‐osmolality (from a cartilage disk) served as the controls to check for possible effects of osmolality on cross‐linking process. The vials containing the samples were then placed in an incubator at 37°C for 96 h to induce cross‐linking of the collagen fibrils (Fig. 1).

### Micro‐Indentation

Before and after incubation with l‐threose, cartilage quarter disks were allowed to reach equilibrium at 400 mOsm/kg water enriched with protease inhibitor for 1 h at 4°C, while glued (5,400 ergo, Germany) to the bottom of a petri‐dish with the cartilage surface facing upward. Therefore, indentation was always performed at equilibrium condition to capture the differences due to cross‐linking only. Using a permanent marker with fine tip, a reference point was specified on the cartilage surface as the starting point of the micro‐indentation process. Micro‐indentation was performed following the protocol determined in a previous study^14^ within a 1.5 × 1.5 mm^2^ area on the cartilage surface using an array of 81 equally spaced (9 × 9) contact points (Fig. 1). For this purpose, a displacement‐controlled indenter (Piuma, The Netherlands) was used which consisted of a controller, optical fiber, and spherical probes with diameters of ∼100 μm and stiffness values of ∼50 N/m (Optics, The Netherlands). The force‐displacement curves were obtained after indenting each contact point with the applied indentation depth (Piezo movement) of 18 μm. The actual indentation depth in the cartilage tissue varied based on the stiffness of each contact point, which can be calculated by subtracting the cantilever deflection from the Piezo movement. Indentation protocol at each contact point consisted of three consecutive steps of loading with Piezo movement of 18 μm for the second, holding time of 7 s, and unloading for 20 s. The effective Young's modulus at each contact point was calculated based on the Oliver–Pharr theory, that is, calculating the slope of the initial portion of the unloading curve. See Moshtagh et al.^14^ for a more detailed description of this optimized indentation protocol.

For each sample, the average of the Young's modulus in 81 contact points was calculated before and after incubation in l‐threose. The difference between the moduli measured before and after the cross‐linking process was used as a measure of the cross‐linking efficiency:

(1) $$\text{Change in effective Young"s modulus (\%)}=\frac{E_{after}-E_{before}}{E_{before}}\times 100$$

where *E_after_* and *E*_*before*_ are, respectively, the effective Young's moduli after and before incubation in l‐threose.

### Contrast Enhanced micro‐CT

To check the gained negative electric charge of the tissue matrix following incubation in the l‐threose solution, the samples were scanned with micro‐computed tomography (Quantum FX, Perkin Elmer, Waltham, MA) at 90 kV tube voltage and 180 μA tube current and voxel size of 20 μm^3^ after 24‐h incubation in Hexabrix solution (40 v/v% GE Healthcare, The Netherlands, 320 mgI/ml, MW = 1269 g/mol, charge = −1) enriched with Protease inhibitor (cOmplete, Roche, Mannheim, Germany) before and after incubation with l‐threose. To eliminate the possible effects of Hexabrix on the cross‐linking process, samples were washed out in a saline bath (400 mOsm/kg water, protease inhibitor) according to a previous study.^15^ The average of the grey scale values was calculated in 10 mid‐slices using imageJ (public domain, NIH, version 1.47).

### Assessment of Color of Cartilage Surface

Depending on the intensity of the cross‐linking, that is, ageing, the color of cartilage undergoes alterations from white (normal cartilage) to brown (cross‐linked cartilage).^16^ To quantify possible color changes as an indication of the cross‐linking following incubation with l‐threose, samples were placed side‐by‐side and digital images from the cartilage surface were captured from above (Samsung Galaxy S6, 12 Megapixels) at the same time during the day. The resulting RGB images were then converted to 32‐bit images and the average pixel intensity was calculated with imageJ.

### Collagen and Pentosidine Content

Each quarter disk was first weighed before freeze‐drying (Christ alpha 1–2 LD plus) for 48 h. The dry weight of the samples was measured after freeze‐drying. 800 μL HCL (Sigma–Aldrich) was added to the dried samples before placement in screw cap vials and subsequently in an oven at 95°C. After 20 h, the liquid phase of the digested samples was allowed to completely evaporate at 60 °C under a fume hood. After adding 500 μl mQ water to the vials, they were vortexed, centrifuged, and prepared for hydroxyproline measurement. Measuring the hydroxyproline allowed for calculating the collagen content of each quarter disk. The complete procedure for the hydroxyproline assay can be found elsewhere.^17^

Pentosidine cross‐linking level within the digested samples was determined based on high performance liquid chromatography (HPLC).^18^ The peak representative of the pentosidine cross‐links was found for all samples, while care was taken to exclude the interference of other peaks as much as possible.

### Statistical Analysis

Data distribution was checked based on Kolmogorov–Smirnov normality test using GraphPad, Prism 5. For data passing the normality test One‐way ANOVA was used. For data not passing the normality test, Kruskal–Wallis was used and *p*‐values less than 0.05 were taken as indicators of statistical significance.

## RESULTS

### Micro‐Indentation

**Figure 2:**
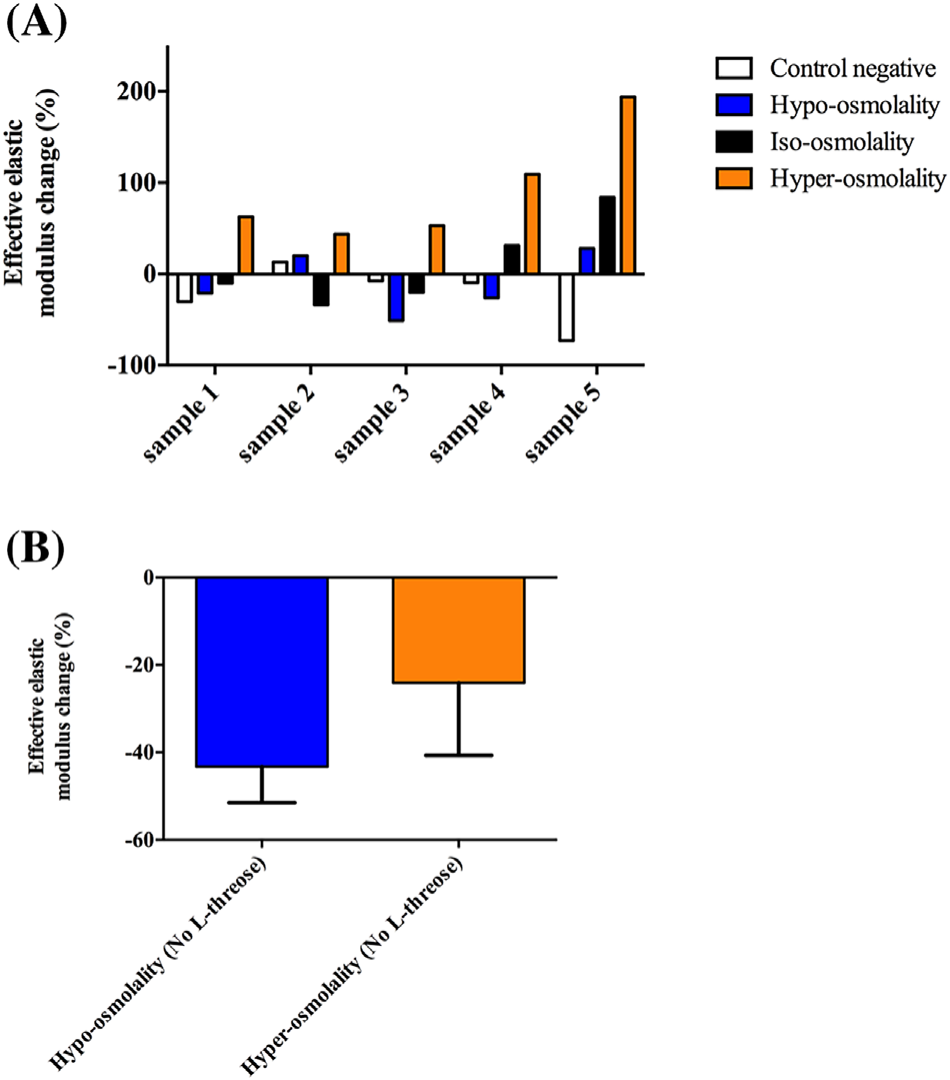
(A) Indentation was always performed at equilibrium condition to capture the differences due to cross‐linking only. Alterations in the effective Young's modulus following the incubation with l‐threose compared to sample's intrinsic effective Young's modulus before incubation with l‐threose. The change of effective Young's modulus of hyper‐osmolal versus control negative as well as hypo‐osmolal versus hyper‐osmolal was statistically significant (*p‐*value = 0.005). (B) Effective Young's modulus change (%) before and after incubation in Hypo‐ and Hyper‐osmolality solutions without l‐threose. The term “before” represents the measurement under the normal osmolality and the term “after” represents the measurement again under normal osmolality but after exposing the sample to hyper/hypo‐osmolality. These tests were conducted to check the possible effect of osmolality on the cross‐linking even in the absence of l‐threose.

The effective Young's modulus increased after cross‐linking, as captured using micro‐indentation before and after cross‐linking with l‐threose (Fig. 1). The change in the effective Young's modulus (Equation (1)) was −21.5 ± 32.6% for non‐treated specimens, 10.0 ± 33.2% for hypo‐osmolal specimens, 10.1 ± 47.9% for iso‐osmolal specimens, and 92.5 ± 62.1% for hyper‐osmolal samples. Our results showed that within each group, the effective Young's modulus was substantially higher for cartilage specimens that were exposed to high osmolality (shrunk samples) during the l‐threose treatment as compared to those exposed to either low or iso‐osmolality conditions (Fig. 2A). Moreover, a decrease in the effective Young's modulus of non‐treated samples was observed. The effective Young's modulus of the non‐treated samples decreased 27.2% for the hyper‐osmotic group and 45.5% for the hypo‐osmotic group suggesting only negligible chemical effect of osmolality itself on the cross‐linking (Fig. 2B). The details of micro‐indentation data per each group and sample are provided as a supplementary file (Figs. S1 and S2).

### Contrast Enhanced micro‐CT

**Figure 3:**
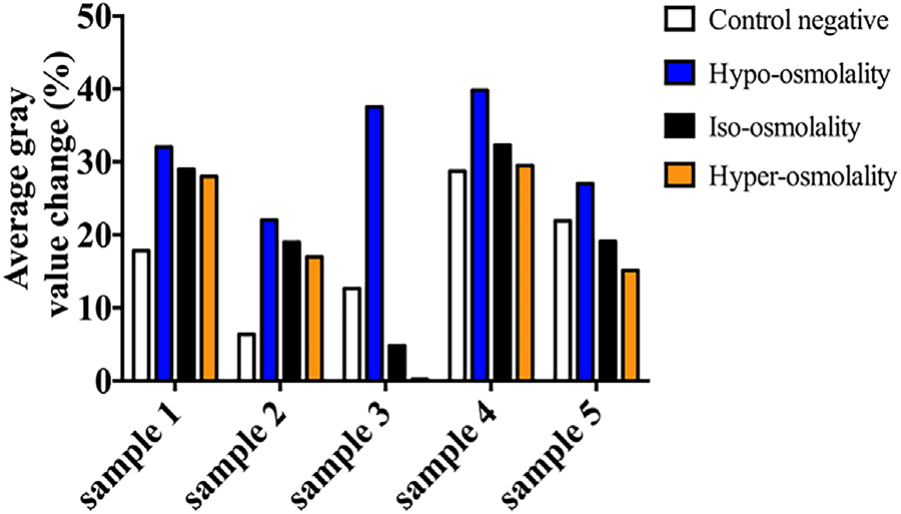
Increase in average grey value indicating the Hexabrix penetration depending on the net negative charges in the ECM. The equilibrium penetration of Hexabrix is inversely related to the amount of matrix fixed charge density.

Previous studies showed that cross‐linking is associated with the formation of free negative groups on the collagen molecules, which leads to increased density of net negative charges within the extracellular matrix.^19^ Determination of possible increase in the negative charge within the extracellular matrix was achieved using contrast‐enhanced micro‐CT. However, increase in negative charge density due to cross‐linking was counteracted by possible leakage of negatively charged GAGs due to enzymatic activity within the matrix particularly during prolonged incubation with l‐threose. Our experiments on the negative control samples (iso‐osmolality and no l‐threose) confirmed tangible GAG loss in those samples (Fig. 3). Therefore, average grey values obtained before and after incubation with l‐threose indicate the effects of both aforementioned phenomena. The increased penetration of Hexabrix followed a rising trend toward decreasing the osmolality within each group of samples (Fig. 3, *p*‐value = 0.81).

### Assessment of Color of Cartilage Surface

**Figure 4:**
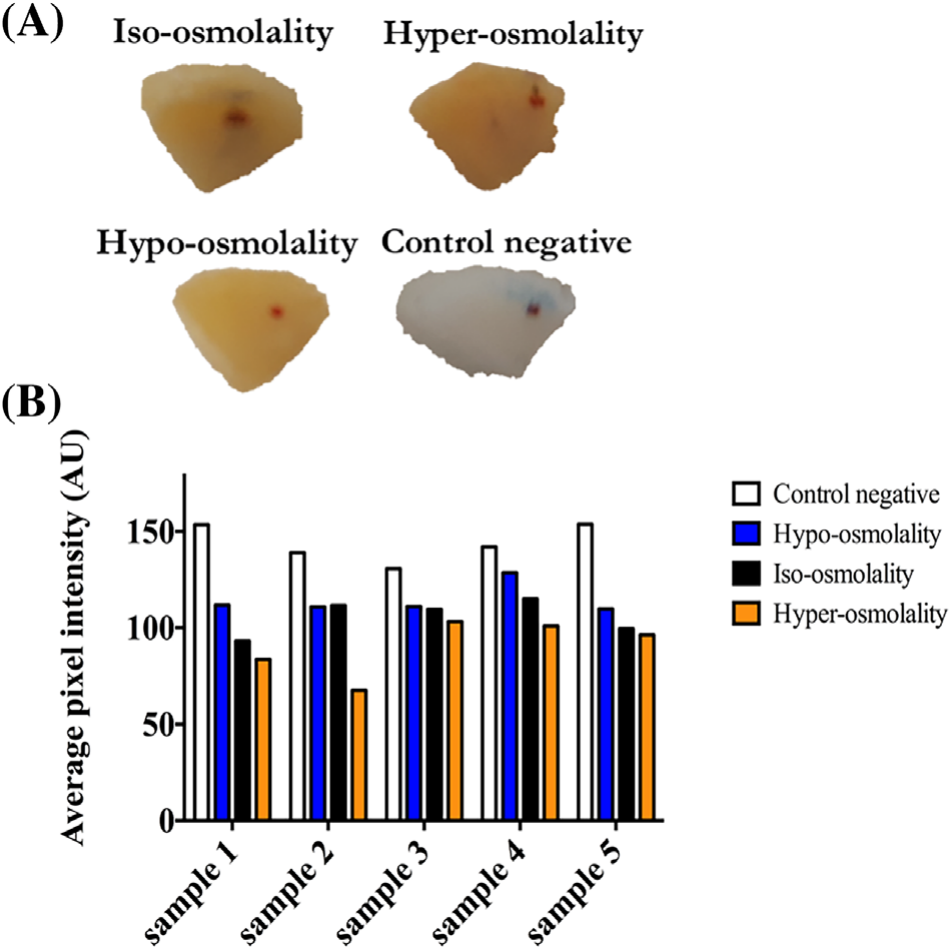
(A) Changes in the color of horse cartilage for negative control, iso‐osmolal, hypo‐osmolal and hyper‐osmolal specimens after 4 days incubation. (B) Cartilage surface color analysis: Brighter color represents higher average pixel intensity, while dark yellow/brown represents lower average pixel intensity (*p*‐value < 0.005).

Non‐enzymatic cross‐linking caused the white surface of normal articular cartilage to turn brown. Our cartilage surface color analysis allowed us to clearly identify differences in the intensity of the brown color between treated and non‐treated normal specimens. The intensity of the color was an indicator of efficacy of the cross‐linking process as the trend suggests (Fig. 4).

### Pentosidine Level

**Figure 5:**
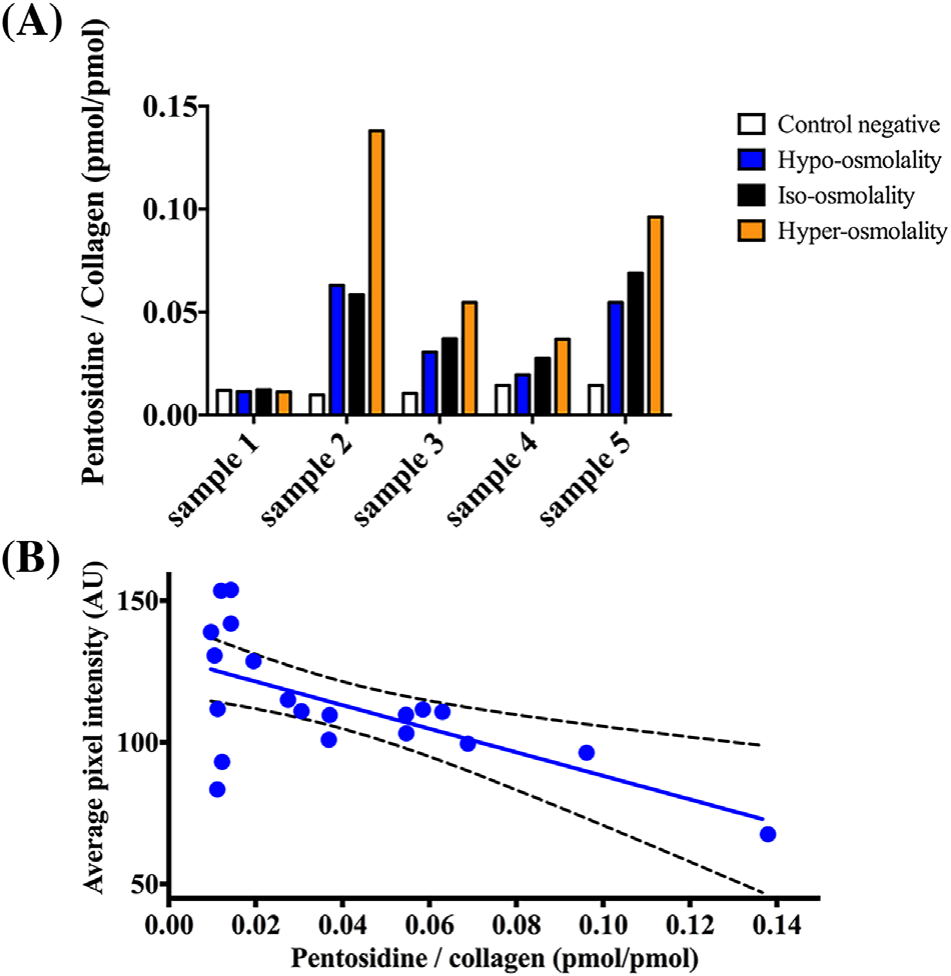
(A) Amount of pentosidine per collagen (mol/mol) is reported for different osmolality conditions. In average, the hyper‐osmolality led to higher accumulation of pentosidine per collagen molecule (mol/mol) (*p*‐value > 0.05). The difference was higher between hyper‐osmolality and control negative (*p*‐value = 0.067). (B) Average pixel value of the cartilage surface versus= pentosidine per collagen molecules (*r* = −0.63 and *p*‐value = 0.0027).

In general, the pentosidine level per collagen (pmol/pmol) increases with the level of osmolality during the l‐threose treatment (Fig. 5A). The amount of pentosidine per collagen (mol/mol) increased 47% from hypo‐osmolality toward hyper‐osmolality (average difference over the five samples). Plotting the amounts of pentosidine per collagen molecules versus surface color revealed a decreasing trend as expected (Fig. 5B).

## DISCUSSION

We investigated the effects of the mechanical strain experienced by collagen fibrils on their chemical response to non‐enzymatic glycation, leading to pentosidine cross‐linking. Due to the fixed charges of the glycosaminoglycan molecules enmeshed within fibrillary network of collagen molecules, articular cartilage allows for the exchange of water from/to the external bath. To maintain osmotic balance, normal articular cartilage swells when exposed to hypo‐osmolality, while it shrinks under hyper‐osmolality. Different joint diseases have been shown to cause a decrease in the osmolality of the synovial fluid, particularly in osteoarthritic and rheumatoid arthritis.^20^, ^21^ In this study, osmolality was adopted as a means to induce expansion and shrinkage of the articular cartilage and its collagen fibers. Measurements of the effective Young's modulus of cartilage (stiffness) at several locations within a prescribed indentation matrix before and after l‐threose incubation were performed. Our data indicated that incubation with l‐threose as the reducing sugar results in stiffened cartilage matrix, which agrees with the findings of a previous study.^22^ Our micro‐indentation data illustrated that l‐threose treatment under hyper‐osmolality enhances the formation of advanced glycation end‐products (Fig. 2). In contrast to hyper‐osmolality, hypo‐osmolality showed restrictive effects on the non‐enzymatic glycation cross‐linking, indicating that expanded collagen fibrils are less susceptible to react with l‐threose. Our observations are in line with findings of previous research that underscored the importance of stretching the collagen molecules in various forms of molecular, fibrillary and tissue level, to protect them against enzymatic activity.^7^, ^8^, ^9^, ^10^ Sufficient compressive strain of the extracellular matrix, that is, 25% strain, has been shown to buckle the collagen fibrils^23^ and consequently affect the conformation of collagen building blocks, that is, amino acids, which is believed to affect the susceptibility to enzymatic degradation. Our micro‐indentation data suggest that expansion protects the ECM against glycation or the so‐called ‘artificial ageing’, whereas shrinkage makes it prone to glycation, although in our study the induced shrinkage and expansion were below 25% of the original cartilage thickness. It should be noted that due to heterogeneous distribution of cartilage constituents, that is, GAGs, collagen fibrils and water content, the swelling behavior is different among the specimens which affects the cross‐linking efficiency. This explains the degree of variation of the changes in mechanical properties of the matrix, nevertheless, in the current study indentation at multiple locations allowed capturing those alterations accurately. As the main direction of swelling/shrinkage was perpendicular to the cartilage surface, the collagen fibrils located in the deeper zones are potentially affected to a larger extent by cross‐linking. The micro‐indentation performed here mainloy captures the effects of the cross‐linking in the superficial zone, however, deep indentation would allow capturing the effects in the deep zone of cartilage. Although it is expected that induction of cross‐linking affects both bending stiffness and tensile stiffness, we mainly measured the bending stiffness in this study. Additional tensile stiffness tests could therefore be proposed to expand the mechanical data. Using a bath containing a neutral contrast agent (*iodixanol*), we determined the swelling behavior of equine cartilage at the osmolalities applied in this study when articular cartilage was kept attached to the subchondral bone. Those experiments showed; post free swelling, less than 4% at hypo‐osmolality (100 mOsm/kg water) which is consistent with a previous study,^23^ whereas hyper‐osmolality (4000 mOsm/kg water) resulted in less than 3% shrinkage (data not shown). This infers that relatively mild matrix deformations are enough to affect the cross‐linking efficiency. Moreover, it is well established^23^, ^24^ that high salt concentration in the bath modulates up ions flux through the cartilage matrix, shielding the negatively charged proteoglycans from further ionic interactions, which results in more relaxing state of the collagen fibrils (less pre‐stress). In addition, higher ionic concentration in the extrafibrillar region than the intrafibrillar region expels the intrafibrillar water ^23^, causing denser collagen fibril network (crushed collagen fibrils), which obviously alters the spatial conformation of the lysin and arginine, mainly responsible for non‐enzymatic cross‐linking. Moreover, the intrafibrillar water normally follows an exponential decay function of the applied external osmotic pressure ^23^, which has likely influenced our cross‐linking efficiency the most.

Previous studies have shown that the osmolality of the synovial fluid decreases in the patients with osteoarthritis and rheumatoid arthritis.^20^, ^21^ As this will create increased swelling of the cartilage (in an early phase of the disease) it might be a protective strategy from advanced glycation in the diseased joint. Moreover, exercise has been shown to be associated with decreased osmolality of the synovial fluid.^25^ Our data, although not exactly in‐line with the physiologic osmolalities, suggest that by maintaining the osmolality of the environment surrounding the cartilage sufficiently yet wisely low, the chance of cross‐linking of the collagen fibrils can be minimized.

It is well‐known that non‐enzymatic glycation results in accumulation of net negative charge generated by the additional group on the collagen molecules.^19^ To identify increased net negative charge post l‐threose treatment, negatively charged contrast agent (Hexabrix) was used, which its equilibrium concentration inversely correlates with the amount of formed negatively charged groups. The penetration of Hexabrix was shown to follow an osmolality‐dependent trend (Fig. 3) which means higher osmolality results in less penetration of Hexabrix, likely due to direct electrostatic interaction between glycation‐driven negatively charged groups and Hexabrix. Furthermore, in highly cross‐linked collagens, steric hindrance, which slows the diffusion rate down, also plays a role as a barrier against Hexabrix transport.^15^, ^19^

The color of the cartilage surface undergoes a shift from white toward yellow/brown in non‐enzymatic cross‐linking.^26^ Therefore, the pixel intensity of cartilage surface could provide potential indication of the intensity of the cross‐linking process. The pixel intensity of cartilage surface was significantly different between the control and other samples indicating the efficacy of the cross‐linking process. It varied according to the osmolality of the external bath with the highest difference observed between the hypo‐ and hyper‐osmolality conditions among all l‐threose treated samples (26.6% based on hyper‐osmolality). Similar to the observations regarding the surface color, pentosidine per collagen molecule values also confirm that increasing the osmolality leads to increased non‐enzymatic cross‐linking (Fig. 5A).

We have found that hypo‐osmolality and the related stretching of collagen molecules, lowers the glycation (ageing) efficiency in collagen fibrils. We believe that this is due to the micro‐unfolding of the triple helix.^27^ Another study suggested that cross‐linked collagen fibrils are more susceptible to enzymatic degradation when stretched in tendon tissue,^28^ which again sheds light on the importance of underlying forces that regulate chemical processes in connective tissues.

It is widely believed that the superficial layer of articular cartilage represents a highly inhomogeneous distribution of collagen fibrils and glycosaminoglycans, which creates heterogeneous mechanical behavior.^1^ Due to this fact, slight differences between iso‐osmolality (400 mOsm/kg water or 0.2 M NaCl) and hypo‐osmolality (100 mOsm/kg water or 0.05 M NaCl), which are relatively similar conditions as compared to the hyper‐osmolality condition (4,000 mOsm/kg water or 2 M NaCl), may not be fully captured. The elastic properties calculated here indicate the pre‐stress in the collagen fibrils,^14^ but as articular cartilage is intrinsically a poroelastic material its hydraulic permeability could provide additional information about the possible restrictions against fluid flow due to cross‐linking. Freezing post‐harvest could potentially affect the organization and subsequently function of collagen fibrils, however, our preliminary study measuring the mechanical properties of cartilage confirmed that freezing the cartilage specimen post‐harvest in the absence of liquid at −20°C minimizes the detrimental freezing effects on collagen fibrils.

As cartilage properties changes across its thickness, one could apply deep indentations to obtain details regarding depth‐wise mechanical and physical properties. Solute features such as permeability through extracelluar matrix could also affect the cross‐linking efficacy as accumulation of small ions in the extrafibrillar region causes exudation of the intrafibrillar water, which potentially restricts the accessibility of l‐threose to the collagen molecules and is therefore worth investigating.

## CONCLUSIONS

In conclusion, we have shown that non‐enzymatic cross‐linking of collagen fibrils of articular cartilage can be controlled through shrinking or stretching of the cartilage tissue, which we applied through adjustment of the bath osmolality. Our micro‐indentation data, assessment of color of cartilage surface, pentosidine level measurement, and contrast‐enhanced computed tomography data all show that increased osmolality accelerates advanced glycation and the “ageing” of articular cartilage. Our findings contribute towards understanding how the mechanical environment of the articular cartilage influences the chemical reactions between sugars and collagen building blocks at micro‐scale, which undergo micro‐unfolding of the triple helix in the hyper‐osmolal and glycation sensitive conditions. We believe that more advanced understanding of collagen fibrils mechanochemistry provided here, evokes potential ageing prohibiting strategies against cartilage deterioration.

## AUTHORS’ CONTRIBUTIONS

BP has substantial contributions to conception and design, acquisition and interpretation of data, and drafting the manuscript; PRM contributed to acquisition and interpretation of data; VA contributed to interpretation of data; JS and RS contributed to acquisition of data; JR, JM, AAZ contributed to interpretation of data and drafting the manuscript; and HW contributed to conception and design, interpretation of data, and drafting the manuscript.

## ETHICAL APPROVAL

Equine knees for this study were obtained from the Equine Clinic in Utrecht University, approved by *Animal Experiments Committee in Utrecht University*.

## Supporting information

Additional supporting information may be found in the online version of this article at the publisher's web‐site.

Supporting Data S1.
