# Supplementary material for: Non‐enzymatic cross‐linking of collagen type II fibrils is tuned via osmolality switch
Source: J Orthop Res. 2018 Feb 13;36(7):1929–36. doi: 10.1002/jor.23857 (PMC6099510; doi:10.1002/jor.23857)
Supplement: Supplementary file 1 — Supporting Data S1. [file JOR-36-1929-s001.doc]

**Supplementary data**

The micro-indentation data for each treatment group (Figure S1) and for each sample (Figure S2) are provided as follows:


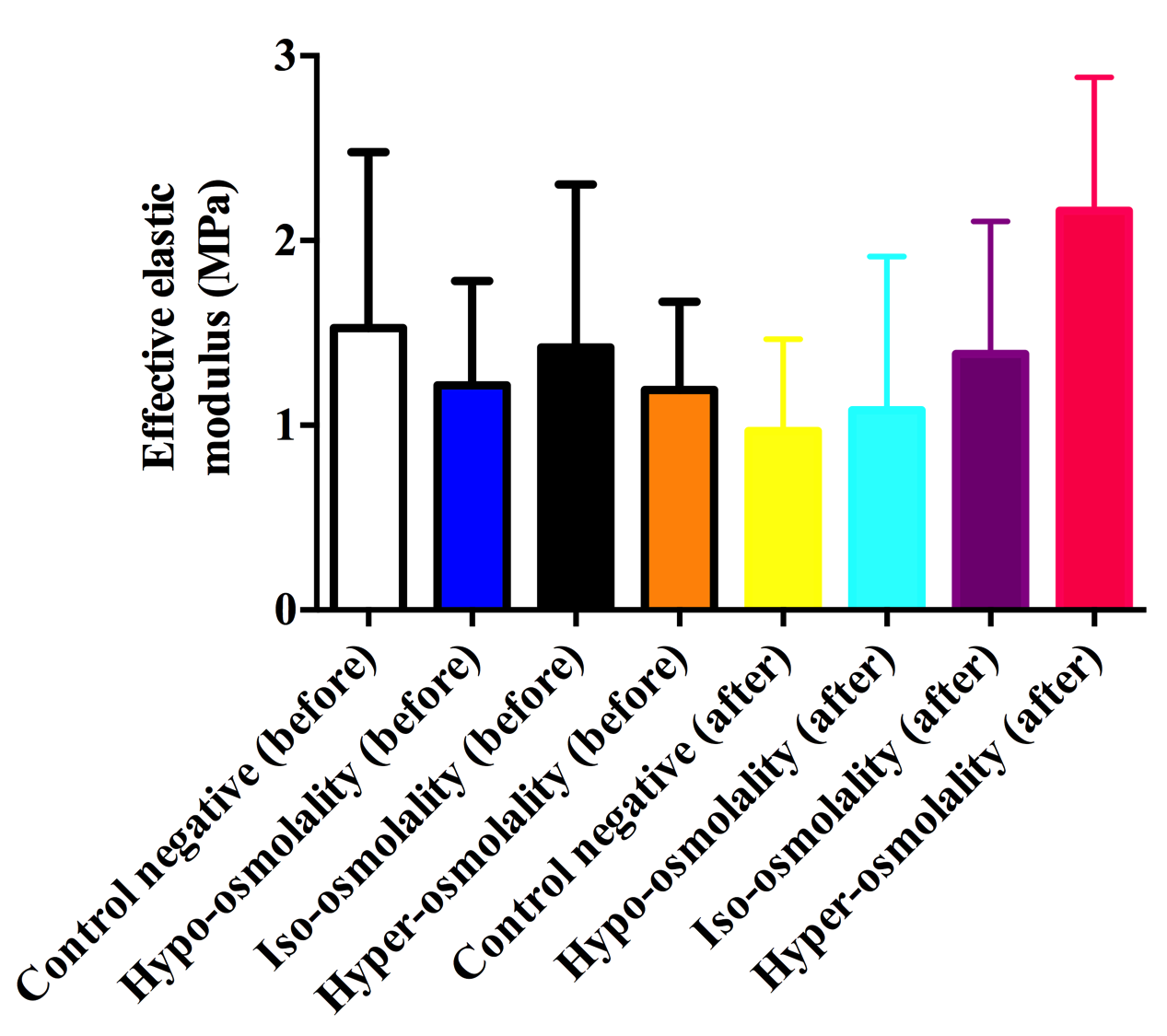


Figure S1. Effective elastic modulus (MPa) for different groups before and after treatment with L-threose.


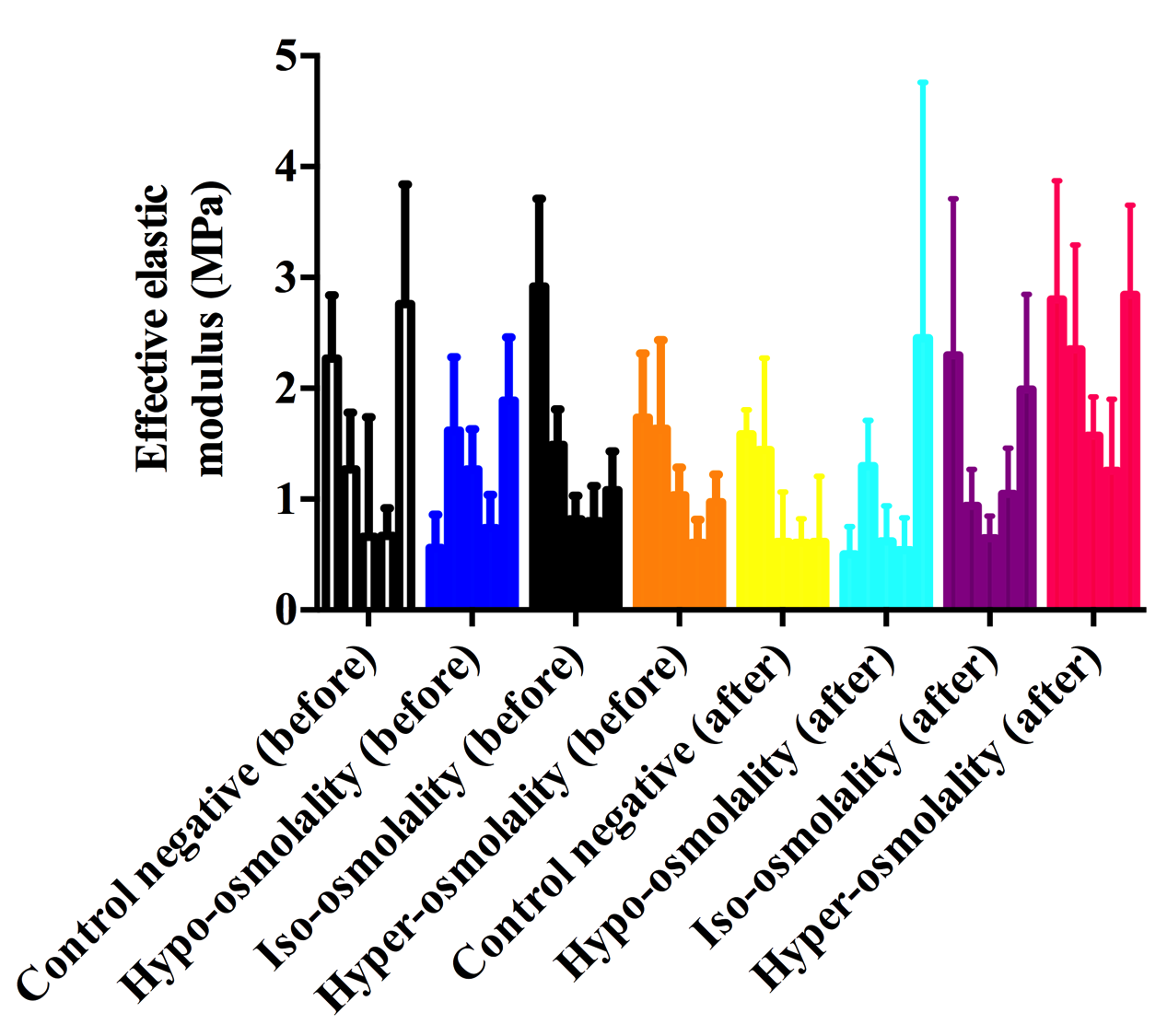


Figure S2. Effective elastic modulus (MPa) for different samples before and after treatment with L-threose.
